# Economic and technical aspects from biomarker identification to companion diagnostic - Co development

**DOI:** 10.1186/1878-5085-5-S1-A101

**Published:** 2014-02-11

**Authors:** Kurt Krapfenbauer

**Affiliations:** 1 University of Vienna, Austria

## 

In view of an urgent need for novel therapeutics that have a positive impact on morbidity and mortality of chronic diseases, the field is now moving more quickly towards clinical translation from biomarker identification to the development of an companion diagostic (CDx) test. The implimentation of such CDx test is driven by smart preclinical validation, a better study design and improved surrogate readouts using currently available methodologies and diagnostic techniques. Moreover, upcoming novel biomarkers and diagnostic technologies will soon permit a more accurate and efficient assessment of disease progression and regression. The ability of biomarkers to improve treatment and reduce health-care costs is potentially greater than in any other area of current medical research. For example, the American Society of Clinical Oncology estimates that routinely testing people with colon cancer for mutations in the *K-RAS* oncogene would save at least US $600 million a year. On the other side, many papers in the course of biomarker discovery projects have been published yet, but only few clinically useful biomarkers have been patented and successful validated for routine clinical practice. The reasons for the limitation to bring biomarkers successful on the market are manifold. Regulatory and scientific hurdles as well as lack in the biomarker validation and lack in the technical validation have been cited as one of the main reasons for the slow growth in this area. Whilst the majority of the published biomarkers will not be translated into diagnostic tests, the growth of biomarker used in clinical diagnostics indicates that the future of the industry will lie in the CDx co-development. The first drug introduced using the CDx co – development – Herceptin (trastuzumab; Roche/Genentech) – has now been on the market for several years. Our study focus on some economics and technical factors that should be considered to successfully develop and market a companion diagnostic test, based on lessons learned from recent case studies. A proposed framework of considerations to be addressed at the various stages of developing effective companion diagnostic test is also presented.

